# Indigenizing wild animal sovereignty

**DOI:** 10.1111/josp.12498

**Published:** 2022-12-09

**Authors:** Dennis Papadopoulos

**Affiliations:** ^1^ Unit of Ethics and Human‐Animal Studies Messerli Research Institute, University of Veterinary Medicine Vienna Vienna Austria

**Keywords:** animal ethics, comparative philosophy, grounded authority, indigenous philosophy, wild animals

## 1 INTRODUCTION

I encountered a turtle midway through crossing the road. I stopped the car and waited for her, but she had seized up. I got out and gently lifted her to the side of the road. It was a face‐to‐face encounter with a *wild* animal who had unknowingly entered a “human” world. Her action disrupted my naive attitude that a road is a place for me to drive along, a place for cars, and not a place for turtles. But, she just needed to get to the other side; the road cut through her world. My naive attitude that the road is not a place for turtles fails to acknowledge the turtles' jurisdiction over their habitat on both sides of the road. In this article, I explore how Indigenous political ontology, from the First Nations^1^ of Canada and the northern United States, allows us to conceive of a world where animals have jurisdiction over their land. On such an account when roads or other interventions cut through their territories without providing accommodations we have done something wrong.

Wild animals have their place in the world as part of autonomous communities outside human institutions like industrial agriculture, laboratories, zoos, and our homes. In order to restrain human interventions in the places and practices of autonomous nonhuman animal communities, some have suggested that wild animals be understood as “sovereign” (Donaldson & Kymlicka, 2011; Goodin et al., 1997). Designating wild animals “sovereign” is one way to establish the jurisdiction of nonhuman animal communities. In line with the norms of international relations, recognizing wild animal communities as sovereign limits foreign (in this case, humans and domestic animals) access to their spaces and establishes limits on the human ability to intervene when it affects their jurisdiction.

A sovereignty conception of jurisdiction is missing something, namely that wild animal communities have no sovereigns—there are no kings of lion prides, ministers of owl parliaments, or presidents of salamander congresses. Wild animals can only be “sovereign” through human institutions. Recommending a novel institution fails to capture the jurisdiction of wild animals that goes unrecognized when humans fail to appropriately limit their interventions. After all, some humans already advocate for limited interventions in wild animals' territory, for example, when activists and environmental government agencies challenge the construction of highways through wetlands or when Indigenous water protectors and land defenders refuse to allow oil pipelines (Sainato, 2021) or the destruction of old‐growth forests (Larsen, 2021). These advocates are usually not defending the supposed rights of a sovereign nonhuman animal community. Instead, they often defend the rights of First Nations to govern land shared with more‐than‐human beings.^2^

The shared jurisdiction, attested to in some traditional Indigenous thought, offers a paradigm for understanding what jurisdiction nonhuman animals already have. There are two sides to this sharing. First is valuing and preserving ecological relationships, central to a wide range of Indigenous thought and widely advocated in western environmental philosophy (Callicott, 1982, 2000; Leopold, 1949; Rolston, 1988). Second, sharing jurisdiction implies that nonhuman animal communities also have jurisdiction. The latter is the jurisdiction referred to in Anishinaabe stories of negotiating and creating treaties with nonhuman animal communities (Simpson, 2017). I will explain how we might conceptualize this shared jurisdiction as an indigenized version of Wild Animal Sovereignty.

Section 2 outlines how sovereignty as a jurisdiction might extend to wild animals. In Section 3, I contrast Wild Animal Sovereignty with what Pasternak (2017) calls Grounded Authority in her description of traditional Algonquin jurisdiction. I conjecture that Grounded Authority can describe wild animals' jurisdiction bottom‐up from their ecological relationships rather than establishing jurisdiction top‐down from a “sovereign.”

There are three layers of justification behind Grounded Authority. In Section 4, I explain how ecosystems give jurisdiction to multiple communities who must share that land. In Section 5, I explain that this jurisdiction relies on a continuous practice of reciprocal respect, where shared ecological gifts are reciprocated by respecting the others (rivers, nonhuman animals, spirits, etc.) who give them. Such reciprocal respect is practicable by more‐than‐human beings. In Section 6, I address a central objection that interspecies relations may be fraught with hunting or predation that may seem contrary to respect, ordinarily understood. Finally, in Section 7, I consider how humans may negotiate treaties that limit their authority, such that we do not wrongfully monopolize gifts given to more‐than‐human beings. The result is an account of Indigenized political ontology that describes nonhuman animals' shared jurisdiction with humans.

## 2 TURNING TO WILD ANIMAL SOVEREIGNTY

Since the political turn in animal rights, political theorists (Hadley, 2005; Nibert, 2002; Nussbaum, 2006) have started to explore how we should construct a political theory that includes nonhuman animals. The political turn is a complementary project to traditional animal rights projects fighting for minimum standards of ethical treatment (Regan, 1985; Singer, 1974). The political turn asks us to go beyond established structures that might protect wild animals and ask, “how those structures, institutions, and processes might be transformed to secure justice for both humans and animals” (Cochrane et al., 2018, p. 273).

One such political structure, which might secure justice for wild animals specifically, is “sovereignty.” Goodin et al. (1997) suggest that it is arbitrary to exclude great apes from sovereignty, where “sovereignty” is understood in the traditional Westphalian sense—independent nations have absolute control within their borders. The Westphalian system aims to understand sovereignty as the sole and absolute authority of a community's leaders within their borders (p. 827). Goodin et al. (1997) explain that the requirements of the Westphalian system are so minimal that it is arbitrary to exclude great apes. Like humans, great apes have communities that exercise authority over a distinct territory. Therefore, nonhuman great apes are wrongly excluded from the global political community.

A problem with their view is that the legitimacy of the great apes' claim to sovereignty rests upon similarity to humans. Our understanding of sovereignty takes the human case as paradigmatic. Animal communities without a distinctive hierarchy and permanent territory may not fit this well. That is to say, Goodin et al. (1997) are still reifying an anthropocentric and plausibly Eurocentric view while challenging the speciesist exclusion of great apes.

Instead of attempting to show that animal communities are sufficiently similar to this distinctly European and human version of sovereignty, we should recognize that the motivation for extending sovereignty to wild animals is something moral. The inconsistent application of the Westphalian system is not a problem that animal ethics faces. The vulnerability that comes from not fitting in with that system is the issue we must rectify. Donaldson and Kymlicka's (2011) version of Wild Animal Sovereignty^3^ focuses more directly on this vulnerability. They suggest that wild animal communities have an interest in autonomy, and this interest sets the scope of rights attributed by Wild Animal Sovereignty. Donaldson and Kymlicka explain the relationship between an interest in autonomy and territory by saying:

In short, when evaluating whether and how to accord rights to sovereignty to particular communities, what matters is not the legal institutions they happen to possess, but rather whether they have interests in autonomy, which in turn, depends on whether their flourishing is tied to their ability to maintain their modes of social organization and self‐regulation on their territory. (Donaldson & Kymlicka, 2011, p. 173).

This emphasizes that nonhuman animal communities must be able to self‐regulate their territories, but do not need the legal trappings of sovereignty, including sovereigns. Wild animals have attachments to specific places where they might nest, mate, migrate, forage, and forth, and access to the use of these places for their reasons is necessary for that local population to flourish. In this sense, even animals who often live alone or only in pairs may mark territory and gather to mate or nest. Through social activities like this, their communal life maps onto specific places and communities need autonomous access to these places and needs those places to remain intact enough that they remain useful to those communities; this is what is meant by maintaining self‐regulation on their territory.

Founding Wild Animal Sovereignty on interests is a departure from a Westphalian account, wherein appropriate authorities with clearly demarcated territories are the sovereigns with jurisdiction. Instead, Donaldson and Kymlicka are explicitly interested in applying international norms to govern the overlapping jurisdictions of wild animals and humans. In this sense, Wild Animal Sovereignty is only an extension of “sovereignty” in so far as that status invokes international protections; however, Wild Animal Sovereignty may take the shape of a new form of jurisdiction. By distinguishing Wild Animal Sovereignty from Westphalian sovereignty, we also differentiate the establishment of a jurisdiction for wild animals from human communities' struggles for autonomy, self‐governance, and sovereignty.

Even if the jurisdiction of wild animals is not “sovereignty” based on the recognition of legitimate nations and legal systems, calling it “sovereignty” may smuggle in problematic power dynamics. Such a concern is poignantly put by Wadiwel (2015):

Why is it that [advocates of Wild Animal Sovereignty] reproduce elements of existing political structure (citizenship, the nation state and the Westphalian system) which might be open to critical questioning, and why is it that forms of human continuing domination—with respect to key decision making—remain authorised? (Wadiwel, 2015, p. 241).

His specific concern here is that the ability to make decisions, enter into agreements, acquire new property, finance the maintenance of existing property, implement security measures, and so forth, will always be handled by humans. The lack of a sovereign for domestic affairs creates a two‐tiered and overlapping, or over‐reaching, power structure where humans and only humans can do the work of being sovereign.

Inevitably, sovereignty or the overlapping jurisdiction called Wild Animal Sovereignty, leaves a power disparity between wild animal sovereignties and the human sovereignties who might recognize them. The experience of Indigenous communities, in particular, provides an example of the problematic power structures formed by overlapping systems of sovereignty. In Canada, many Indigenous communities are recognized, with some caveats, as sovereign in their own right. Nevertheless, Settler Canada fails to respect Indigenous autonomy, focusing instead on WEIRD (western, educated, industrialized, rich, and democratic) interests. As a result, this overlapping sovereignty system leaves many Indigenous people in situations of insecurity or crisis (Ball, 2021; Drinkwater, 2021; McKinley & McKeen, 2020). This context of ongoing oppression, despite recognition of “sovereignty,” has led to widespread criticism of the politics of recognition (Coulthard, 2014). Alfred (Kahnawake First Nation, 2006) argues:

Sovereignty is an exclusionary concept rooted in an adversarial and coercive western notion of power. Indigenous peoples can never match the awesome coercive force of the state; so long as sovereignty remains the goal of indigenous politics, therefore, Native communities will occupy a dependent and reactionary position relative to the state. (Alfred, 2006, p. 325).

Here Alfred offers one argument against focusing on obtaining sovereignty for marginalized Indigenous communities on western terms (i.e., in a Westphalian model of sovereignty). He is concerned that even when sovereignty is recognized, the power structures at work can maintain colonial domination. He is not discussing animal ethics, however, this passage highlights several features of sovereignty about which we ought to think critically before we endorse extending sovereignty or Wild Animal Sovereignty to nonhuman animals; sovereignty is an exclusionary, adversarial, and coercive power. The recognition or extension of sovereignty to wild animals should not be conceived of as setting up adversarial and coercive sovereigns where wild animal communities will always be less powerful, reactionary, and dependent participants in continuing conflicts. Instead, we want to find an inclusive, nonadversarial, noncoercive way of understanding interspecies shared jurisdiction.

These exclusionary, adversarial, and coercive dimensions of sovereignty need not be part of Wild Animal Sovereignty. Donaldson and Kymlicka (2011) started divorcing their conception from Westphalian sovereignty by rejecting legal institution as the justificatory grounds of Wild Animal Sovereignty. Further, they are expressly interested in the forms of shared jurisdiction central to Indigenous legal traditions, rather than the politics of recognition criticized by thinkers like Alfred (2006). Kymlicka and Donaldson (2014) suggested that animal ethics can be part of a broader challenge to the status quo. Through animal ethics, we should:

de‐center and denaturalize majority practices, open up space for cross‐cultural learning, guard against the instrumentalization of progressive causes, and above all would shine a light on forms of power and privilege that have been immunized from ethical accountability. (Kymlicka and Donaldson 2014, p. 129).

In the name of just such a decentering of WIERD Settler ontologies, Donaldson (2020) suggests Wild Animal Sovereignty attempts to “renew western political thought” by also thinking of “animals as political agents and members of political communities” (Donaldson, 2020, p. 23). In particular, she mentions that in Anishinaabe thought, wild animal communities are conceived of as clans with whom human clans have political relationships. Following their prompt, I want to explore more closely just how the Anishinaabe and other First Nations' ecocentric political philosophies can explain the legitimate jurisdiction of nonhuman animals without the Westphalian attribution of absolute power to a sovereign over a distinctive and exclusive territory. This exploration will Indigenize Wild Animal Sovereignty by outlining an ecocentric kind of jurisdiction.

Before I advocate for views rooted in Indigenous thought, I must make clear that I am not Indigenous. I am a settler who grew up in the area known as Tkaronto (from which Toronto derives its name) it has been cared for by the Anishinabek Nation, the Haudenosaunee Confederacy, the Huron‐Wendat, and the Métis, and is currently home to many Indigenous Peoples. I acknowledge both this history and the current treaty holders, the Mississaugas of the Credit First Nation. This territory is subject to the Dish With One Spoon Wampum Belt Covenant, an agreement to peaceably share and care for the Great Lakes region.

## 3 GROUNDED AUTHORITY

As a starting place for reconceptualizing jurisdiction outside of Westphalian sovereignty, I look to the Algonquin of Barriere Lake. Their political ontology has a form of jurisdiction that is justified partly by the shared flourishing of humans, nonhuman animals, and the ecosystem. Pasternak calls this form of jurisdiction Grounded Authority,^4^ which offers an ecological justification of jurisdiction that does not rely on the legal institutions of a community. I argue Grounded Authority describes the sort of jurisdiction advocates of Wild Animal Sovereignty might want to attribute to wild animal communities; one which shares territory instead of being exclusionary, relies on leaving enough for others instead of being adversarial, and focuses on respect for ecosystems and intercommunity kinship rather than justifying coercive power. This authority is “grounded” insofar as chiefs or landholders are justified, partly, by relationships with and knowledge about that land. The traditional knowledge of the land is kept alive in stories called “Onakinakewin.” A chief has a duty to protect the Onakinakewin, and candidates for leadership are evaluated partially on their knowledge of the land through a process called “blazing,” where they must learn the Onakinakewin from their elders. With this knowledge, a chief is traditionally responsible for the appropriate movement and deployment of people on the land. To fulfill this responsibility, a chief looks to two major considerations. First, distribution depends upon the abundance of the land such that each family can sustainably thrive. Second, the relationships between particular families and the places where those families have traditionally lived and hunted should be respected. These traditional relationships between particular families and places suggest those families have especially careful knowledge of those places—how to live, hunt, and preserve nature there.

In this system, relationships to the land and knowledge of its seasons, plants, and animals are part of the justificatory ground for the political authority of the chief and landholders. Pasternak (2017) explains:

The Onakinakewin exposes the background picture of jurisdiction, […] which is comported in the daily practices of hunting, gathering, speaking Algonquin, and living on Barriere Lake territory. Jurisdiction […] retains its integrity through quotidian land use and stewardship. (Pasternak, 2017, p. 95).

The knowledge and traditions of the Algonquin help sustain relationships with the land and animals who live there. These relationships enable landholders to live off the land; the same knowledge and relationships that enable sustainable living also justify their jurisdiction. This conception of jurisdiction protects both those living off the land and the land itself. When the jurisdiction of the Algonquin is respected, it can lead to more sustainable land management through consultation and partnership (Van Schie & Haider, 2015), as well as greater community well‐being (Fligg & Robinson, 2020).

However, I am not recommending that human communities all adopt Grounded Authority. The focus of this article is not to issue another call for ecologically minded recognition of the needs of more‐than‐human beings. Grounded Authority may be a kind of “land ethic” by Leopold's (1949) account, wherein “A thing is right when it tends to preserve the integrity, stability, and beauty of the biotic community. It is wrong when it tends otherwise” (p. 224). However, Grounded Authority is especially interesting because it justifies jurisdiction through respect for sustainable ecology. Further, in principle, this justification is available to wild animal communities with sustainable ecological know‐how.

Pasternak identifies some of the central knowledge for Grounded Authority in wild animal communities, even though she does not directly suggest that in virtue of this knowledge, wild animals too have Grounded Authority. Pasternak explains that the Algonquin language is conceived of as part of knowing the land, partly because it contains the specific toponymy of the land as well as the names of animals and plants. Identifying salient geography and ecology is key to successful hunting, gathering, and living off the land. This rich knowledge of particular elements of the environment is not limited to humans. Wild animals also know the land, and it is from careful observation of animals that the Algonquins have come to know the land, its toponomy, and edible and medicinal plants. Pasternak recounts:

For example, the Algonquins have observed the beaver uses yellow‐pond lily (*cikitebak*, *akidimô*) for its lungs, the moose uses balsam fir (*aninâdik*) for wounds and sickness […] bears use trembling aspen (*azâdi*) for a spring tonic, laxative, and dewormer. (Pasternak, 2017, p. 87).

This knowledge is captured in Onakinakewin stories and the names of plants and places in the Algonquin language.

Pasternak explains that there is an Algonquin saying that wild animals can speak the Algonquin language because the Algonquin language is the language of the land. She recounts recounting a story where an elder speaks out to some wolves in Algonquin and asks them to leave, and the wolves comply (Pasternak, 2017, p. 96). The transformative idea behind thinking that wild animals can speak the language of the land is that wild animals are conceived of as knowledgeable agents who belong on this land—agents with whom humans must negotiate a sustainable way of life. The idea at work here is related closely to the view that wild animal communities' knowledge, or what is sometimes called animal culture (Brakes et al., 2021), is part of what enables those communities to live on their land. Equipped with this knowledge, we can think of wild animals as also having Grounded Authority and holding the land. This claim goes beyond Pasternak's account of Grounded Authority, which is a conception of human jurisdiction as shared with nonhuman animals. However, an implication of that shared jurisdiction is that others, with whom jurisdiction is shared, also have jurisdiction. This independent sense of jurisdiction is illustrated in Anishinaabe practice of negotiating with nonhuman animal communities, discussed in Section 8. However, there are some missing steps between suggesting Grounded Authority may be a shared form of jurisdiction, and negotiating with nonhuman animal communities.

First, I want to explain that nonhuman animal jurisdiction is not authorized by humans, but by their own relationships to ecosystems; the same sort of relationships that justify human jurisdiction. To fully explain this I need to elaborate on how Indigenous political ontology ascribes agency, and a prelegal authority to ecosystems; this is the task of Section 5. Second, the ecosystem's jurisdiction relies on a condition of respecting the gifts provided. In Section 6, I will explain what this kind of respect means in the human context. However, this tells us little about how that respect operates among nonhuman animals. In Section 7, I introduce a distinction between the individual animals and their community, or spirits, such that the interests of individuals are not necessarily the interests of their communities. This will help us explain how communities of nonhuman animals exercise the appropriate political agency needed to respect the land and enter into negotiations with humans.

## 4 SHARING THE GIFTS OF THE LAND

To better explore how jurisdiction is shared, let us think about what it means to have multiple overlapping jurisdictions in an Indigenous context. The Settler government of Canada has claims to share some of the lands of Canada through treaties^5^ with First Nations. These treaties offer one important way of connecting the British common‐law tradition, which founded Settler Canadian law, to the pre‐existing Indigenous legal traditions. Borrows (Chippewa of the Nawash First Nation) interprets treaties as part of Indigenous legal traditions and, through a resurgence of understanding Indigenous law, aims at reconciling Indigenous and Settler jurisdictions.

Borrows starts with a famous promise from Canadian treaty law: the treaties were to endure “for as long as the sun shines, the rivers flow, and the grass grows” (Borrows, 2018, p. 63). From an anglophone Settler's point of view, this sounds like the treaty should endure forever because the sun will always shine, rivers cannot help but flow, and grass cannot help but grow. Borrows explains that some of the language takes on a different meaning when we consider the grammar of Anishinaabemowin^6^ (the language of the Anishinaabe), wherein ecological features that anglophones would describe as inanimate objects count as animate subjects. The animacy of the world is accompanied by a sense of respect for the animate.

Borrows suggests that when we understand rivers as subjects acting on the world, “so long as the rivers flow” should direct attention to the contributions of a flowing river, the abundance it brings, and the environmental wealth that sprouts at the river's mouth. Here we see something about rivers often missing from a western conception of rivers. As an anglophone, I usually think of rivers as water in motion; the same way water moves through the plumbing to my tap and down my drain; this misses all the other vitality in and around the river.

Borrows (2018) explains that the Anishinaabemowin word for water is *nibi*, related to *nipy*, which means life. The vitality of the rivers and lakes is part of the concept of water itself. Further, the word for the mouth of river *zaagiin* is closely related to z*aagi*, meaning “love” (Borrows, 2018, p. 65). With the animacy and etymology of the river in mind, Borrows suggests the life‐sustaining love of the river ought to be respected since its contributions and abundance are acts of love from an animate subject. Life and love are given to those more‐than‐human communities who depend upon that river. The river does not require life from us; our role in our reciprocal relationship with rivers is to respect them and the gifts they have given. Including the river in the treaty between human societies suggests that the river is a way of understanding their relationship. In addition to respecting the river, we should treat each other with the same love, the same giving without taking, embodied by the river.

This respect is emphasized in Mississaugas^7^ name for themselves. Borrows explains that the word *micha* means large, and *zaagin* means river mouth, so Mississauga means large river's mouth, but it also means place of great love. We might add that in context, the “love” of this place is the river's love.

I grew up in this same place, on both treaty and unceded land of the Mississaugas, where many rivers feed into the great lakes, yet I have always thought of myself as belonging to a civilization that spawned in ancient Greece, spread through the Roman Empire, and finally took over the world through western Europe's imperialist expansionism. However, my body was *given* life by the rivers and the land. I have always thought of wild animals and the land as something we must care for in the spirit of charity or stewardship, but they were still lesser‐than‐human.

If I was taught to appreciate the “gifts” of the land, they were gifts from God (or gods) to humans (or God's chosen people). There was no mention of the activity of the land itself, the gifts rivers give, and the fact that these gifts are given to humans and wild animals. I was taught only to respect the river instrumentally, including its aesthetic values, which still ignores all the other relationships that a river has. It is helpful to recognize the divisive, ecologically naive narrative mythologies of the West, as they may still form a background of intuitions for our more professional meditations.

Changing how we think about land and rivers might change how we think about our relationships with wild animals. My brother was crossing a bridge over the credit river in the city of Mississauga (named after the First Nation whose lands were colonized and settled). From the bridge, he saw a beaver. He noticed a couple walking on the bridge nearby and pointed out the beaver. They responded with disgust and lamented, “that thing will probably go after our trees,” by which they meant the beaver might fell some small decorative trees planted along suburban streets. This sort of thinking fails to recognize that the land is also the beaver's land; those trees are also the beaver's. This land is a gift to all of us (a gift which suburban development disrespects in the first place). Grounded Authority suggests that whatever policies we develop to coexist with animals like the “suburban” beaver must respect that the beaver belongs here too. My suggestion is that when we understand that the “beaver belongs here too,” we attribute that beaver and their community a legitimate jurisdiction, not sovereignty necessarily but a jurisdiction nonetheless.

The prelegal authority of rivers to “give” life and justified jurisdiction goes beyond the intrinsic value western thinkers like Rolston (1988) have attributed to ecosystems. Ecological gifts, like a river's life and love, provide a prelegal sense in which the world is shared. The way that the treaties use references to entities like rivers to show a shared gift from the land to multiple human communities also offers us a way of understanding how the land can be understood to give jurisdiction to multiple interspecies communities.

Rivers are a kind of being that give themselves, and the recipients of that gift all take part in the jurisdiction of that gift. Those recipients owe respect to the river and the others to whom the river gives itself. However, we might distinguish that the river, while capable of giving, may not be capable of respecting, or the sense in which rivers might respect is unlike the sense in which organic beings respect those gifts on which their life depends. Since the gift must be respected for jurisdiction to be legitimate, we will now turn to explain what sense of respect is operating in this type of eco‐centric jurisdiction.

## 5 RESPECT AND RECIPROCITY

The language of respect and reciprocity is common to many Indigenous people throughout North America. It is a core part of many Indigenous moral philosophies involved in the resurgence of Indigenous self‐governance against the Settler governments of Canada and the United States. Beginning with an ontology where the life of all communities is given by the land, rivers, wind, and so forth, and the jurisdiction of communities depends upon their respect for this wellspring, we might now ask what it means to respect the land in such a way that it could inform political practice.

Coulthard (Yellowknives Dene First Nation) describes Indigenous peoples' struggles for self‐governance against colonial and capitalist regimes as:

struggles not only *for* land, but also deeply *informed* by what the land as a mode of reciprocal *relationship* […] ought to teach us about living our lives in relation to one another and our surroundings in a respectful, nondominating and nonexploitative way (Coulthard, 2014, p. 60).

Here, Coulthard contrasts Indigenous self‐governance with the globally dominant colonial and capitalist systems of governance. Thinking of land as both “informative” and part of a “reciprocal relationship” with human societies challenges the European Westphalian “sovereignty” relation where a sovereign has dominion over a land. This sovereign power might be informed and justified by the citizens who voice their collective will, but the sovereign owes nothing, not even respect, to the *land* they rule.

It is important to recognize that even among Indigenous peoples, their philosophy is not always manifest in their political actions. Coulthard examines the historical struggle for Dene self‐governance of the Northwest Territories in Canada. He explains how Indigenous thought informed an initial proposal for self‐governance; this proposal was rejected. Coulthard laments:

a reorientation of Indigenous struggle from one that was deeply *informed* by the land as a system of reciprocal relations and obligations (grounded normativity), which informed our [the Dene] critique of capitalism […], to a struggle that is now increasingly *for* land, understood now as material resource to be exploited in the capital accumulation process (Coulthard, 2014, p. 78).

It is important to recognize that Indigenous land‐informed thinking does not always capture the decisions of Indigenous groups. Further, I am not interested in a historical view of pre‐European Indigenous thought set apart from contemporary practice (see Callicott, 1982, 2000). The unifying feature of Indigenous thought that I rely on common to many thinkers from Algonquin, Anishinaabe, Dene, and other First Nations, is that they all challenge the western ontologies and power structures, manifest in the Settler Canadian exercise of government power. Comparing western political concepts, like sovereignty, to a plurality of different but intersecting ontologies makes space for a reimagining of jurisdiction such that it can be shared with wild animal communities.

Coulthard's ideal challenges the logic of land as a dominion for exploitation by describing how his community conceptualizes “land” differently. He tells us that in the Doghrib^8^ language, “land” or *dè* captures relationships between the material “land,” humans, wild animals, lakes, rivers, and so forth (Coulthard, 2014, p. 60). Understood in this way, humans are part of the land, which includes the whole ecosystem found in that place. Humans have obligations to the land, as a greater whole of which we are just a part, and when we satisfy these, the land reciprocates by providing us with enough. As an example of this entangled set of obligations, Coulthard recounts Elder George Blondin's (Sahtu Dene First Nation) story of Blondin's brother Edward out hunting with a raven.

Edward was hunting near a small river when he heard a raven croaking, far off to his left. Ravens can't kill animals by themselves, so they depend on hunters and wolves to kill food for them. Flying high in the sky they spot animals too far away for hunters or wolves to see. They then fly to the hunter and attract his attention by croaking loudly, then fly back to where the animals are

Edward stopped and watched the raven carefully. It made two trips back and forth in the same direction. Edward made a sharp turn and walked to where the raven was flying. There were no moose tracks, but he kept following the raven. When he got to the riverbank and looked down, Edward saw two big moose feeding on the bank. He shot them, skinned them, and covered the meat with their hides. Before he left Edward put some fat meat out on the snow for the raven. He knew that without the bird, he wouldn't have killed any meat that day. (Blondin 1990, cited in Coulthard, 2014, p. 61).

In this case, the hunter, raven, and moose are all part of the relationships that comprise the land. How the hunter thinks of the raven matters; they are seen as an independent agent, intentionally communicating and giving an opportunity to the human, and the success of this hunt depends on both agents. The relationship between the raven and the hunter is nondominating and nonexploitative because of the interdependence, respect, and reciprocity.

I want to examine what sort of respect and reciprocity is involved here. In this story, western intuitions might track a sense of respect and reciprocity in the relationship between hunter and raven. There is a reciprocal give and take—the raven gives information to the hunter, and the hunter leaves meat for the raven in exchange. The raven is treated as informative and deserving of a choice piece of fatty meat, which shows the hunter's respect for the raven. However, we might be alarmed that those two moose in the story may not be being respected or benefit by the reciprocity at work. Further, it is unclear how the raven shows their respect for either the moose or the hunter. To understand both of these, we will have to explore a division between the individual animal and the ecologically intertwined communities in which they participate.

## 6 INTERSPECIES KINSHIP AND HUNTING

In order to understand what reciprocity means in these cases, we must first explain how these Indigenous concepts of “respect” and “reciprocity” are not identical with their use in western moral traditions. The goal in this section is exegetical; I want to explain what might be meant by the Indigenous accounts of respect, so that we can better understand the kind of agency that nonhuman animals must have to hold a jurisdiction, respect others, and be respected by human communities. My intuition when I hear “respect” in western animal ethics is to think of respecting the rights of nonhuman animals, like bodily autonomy. However, respect for a right to bodily autonomy is incompatible with killing. So “respect” in Indigenous philosophy is not identical to respecting the rights of individuals.

The tension between traditional animal rights and Indigenous moral systems creates a space where animal rights movements may be co‐opted and misconstrued to undermine the autonomy of Indigenous communities (Kymlicka & Donaldson, 2015). To mitigate such conflicts, we may want to highlight the animal ethics discussions within Indigenous communities to understand why respect is morally valuable even if it only partially overlaps with the rights of individual wild animals. For such an exploration, I turn to Robinson's (Lennox Island First Nation) view of Animal “personhood,” which explains that while Indigenous thought may be compatible with sustainable subsistence hunting, outside of that lifestyle (in cities where most Indigenous people live today), decolonizing food practices likely requires abstaining from meat, at least the farmed meats found in our grocery stores. However, her explanation of traditional Mi'kmaq^9^ respect and reciprocity for hunted animals does not rely on something like individual rights even though it arrives at a similar vegetarian conclusion. Stories about hunting relationships can coherently indicate one important sense of respect—respect for our shared ecological relationships.

Robinson recounts a Mi'kmaq creation story. She explains that the creator makes Glooscap a “cultural hero and the archetype of virtuous human life” and his grandmother, Nukumi.

The role of a grandmother is important in Mi'kmaq culture, so much so that Nukumi is the first relative Glooscap acquires. She provides him with wisdom in exchange Glooscap must provide her with food. Nukumi requires meat, for she explains that she cannot live on plants and berries alone (which presumably Glooscap ate before her arrival), so Glooscap calls upon his friend, Apistanewj [which is the Mi'kmaq word for American pine marten]. Glooscap asks Apistanewj, the marten, to sacrifice himself so that Glooscap's grandmother may eat. Apistanewj agrees, and to acknowledge this sacrifice Glooscap makes him his brother. Glooscap breaks Apistanewj's neck and lays his body on the ground. Glooscap immediately regrets his actions, Nukumi intervenes with the Creator, and the marten returns to life. The body of another marten now lies on the ground, available to be eaten without the messy feelings of guilt and loss entailed in the death of friend. In later stories Apistanewj is sometimes described as an animal and sometimes as a human boy, but he is always Glooscap's companion. (Robinson, 2014, pp. 674–675).

To explain Apistanewj's status as simultaneously “dead (and available for eating) and alive (and available for friendship),” or Glooscap's role as both hunter and friend to animals, Robinson introduces a distinction between two ways of referring to animals; there are both individual animals and spiritual beings with whom hunters might form relationships. While Apistanewj—the individual marten—was killed, Apistanewj—the spiritual being or the Marten—lives on and has a relationship with Glooscap. In this way, the Marten can be respected, not as a god who sacrifices individual martens, but as the object of love in a kinship relation between local communities of humans and martens.

### 6.1 Unnecessary spirits objection

I want to take a moment now to address four objections. The first objection is that if respecting animals requires postulating “spirits,” it may be implausible for those committed to the view that there are no such spirits. However, spirits are part of morally salient relationships between communities. These relationships explain the specific sense of respect and reciprocity at work, whether or not we acknowledge these spirits as such.

### 6.2 Abstracted species objection

Second, if some martens are respected and kept well, perhaps in a zoo, then we have respected the Marten and, setting that aside, can now respectfully hunt all wild martens to the brink of extinction. However, the Marten is involved in an active relationship with hunters and is not an abstraction like the species *Martes Americana*, with which humans cannot have an interpersonal relationship. The relationship locates the Marten spirit such that if we were to preserve the species in distant places or zoos, that would not qualify as respecting the spirit of the Marten as it inhabits particular places and particular communities.

### 6.3 Group‐specific interests objection

A third objection is that applying the same logic to human communities would be unintuitive. We would not accept that sacrificing some humans for the benefit of others is permissible, even if we respect the appropriate spirits. However, some martens can be sacrificed for the benefit of humans. More generally, as soon as it is acceptable to harm some individuals to benefit others, we enter into a series of complex problems. Cochrane (2013) raises this general concern against Wild Animal Sovereignty, pointing out that wild animal communities have conflicting interests. We might find that it is in the interest of some wild animal species that we hunt others; for example, the crow has an interest in our hunting moose, and the prey animals, like moose or marten, might have an interest in our hunting their predators, like wolves.

We need to clarify two details to resolve these tensions between group‐specific interests. First, different sorts of ecological relationships between communities require different maintenance. Second, it is the relationship between communities, not the benefits provided by killing one another, which is the valuable thing at issue when discussing wild animal jurisdiction. The Martens as a population can and do tolerate some hunting, just as they tolerate some predation from other wild animals. This tolerance is manifest in their continued flourishing as a community. This “toleration” is not meant to refer to the mental state of some individual martens, rather it is a feature of their population's resilience. Can they, as a community, continue to maintain their way of life in a particular environment despite a threat like a hunter? If yes, then we can say the community tolerates the threat, if no, then that community, either through death or relocation, is not‐tolerating that threat but succumbing to it.

Human communities are not similarly tolerant of predation. The central difference is that the valuable relationships between human communities are more intimate, in order for a human community to respect their relationship with other human communities, they need to do more than maintain enough resources for coexisting populations. So, valuing human relationships with other humans requires not hunting one another. Our relationships with the Martens are not very intimate in most cases, and so those relationships might be respected despite hunting, at least in the context of Apistanewj and Glooscap. In modern contexts, we might argue that we ought to develop the sort of relationship with the Martens or other communities such that hunting them would be a violation of that valuable relationship, and this is a plausible claim. Nonetheless, the object of respect is a relationship, and the participants in that relationship are communities. This is the sort of respect required for eco‐centric coexistence.

We can now see that despite hunting those two moose, the human hunter may still respect the Moose, and more importantly be part of a community which collectively respects the Moose. The individual raven in Blonding's story similarly is participating in respectful relationship with the Moose just insofar as it's behavior is sustainable, ravens do not enable hunters or predators to over‐hunt the Moose. However the human–raven relationship is more intimate than this, that relationship required more, if for example, the raven was not left meat afterwards they would not be inclined to cooperate in the future, damaging that human–raven relationship.

### 6.4 Disrespectful animals objection

The fourth objection suggests that nonhuman animals do not practice this form of respect. The sustainability implicit in what counts as respectful behavior is not obviously something to which nonhuman animals are sensitive. Consider the elk population of Yellowstone, which grew until they began depleting their ecosystem until wolves were reintroduced, and that created a cascade of changes improving biodiversity and controlling the elk population (Boyce, 2018). Those elk did not respect the land.

It is not just other animals who must be respected, but also the land, the rivers, and the plants. The relevant respect maintains the relationships we have with other communities and shows an understanding and appreciation for the gifts of more‐than‐human beings and communities. Robinson (2014) suggests that we can show such respect by never taking more than we need. The Mi'kmaq call this respect netukulimuk or “avoiding not having enough,” we ought to take from the world only what we need to avoid not having enough, just as when we gather, we ought to always leave some significant portion behind for others.

Kimmerer (Citizen Potawatomi Nation) describes respecting the animacy of others in the practice of gathering leeks. She explains that we must not pick the first leek we see and must not collect all of them when we find a patch. Instead, we should respect the population of leeks in the area, leaving an abundance for others, human and animal, this year and for years to come (Kimmerer, 2013, pp. 176–178). However, it is not just the consequence—leaving an abundance—that motivates her respect. It is also respect for the gift of the leeks, which, like rivers, are part of the land given to more‐than‐human beings to share.

In the case of the elk at Yellowstone, we see the land and local interspecies community giving less and less to the elk. Their misuse and disrespect of the land were met with a response suggesting fewer than those high numbers would be tolerated by those with whom the elk must share their jurisdiction. Such cases may warrant the interventions of others, like humans reintroducing wolves.

However, the fact that individual elk lack the intention to leave enough for others does not undermine their jurisdiction. It is the Elk as a community, acting together who have jurisdiction. Individual intention plays a less significant role in this ontology, the river does not intend when it gives, nor do the leeks intend to be our food. The agency or animacy of these entities is responsive to changes in their relationships with others, but they may not have intentions, or their changed activities may not indicate changes in intention. While elk have intentions, it is their behavior that facilitates many of their ecologically important interspecies relationships. Good intentions also have a place in Indigenous moral philosophy, and the distinction between good intentions and respectful land management may be difficult to disentangle in the human case. Nonetheless, we can identify respectful behavior in the ecologically sustainable and knowledgeable behavior of nonhuman animal communities without evaluating their intentions.

## 7 NEGOTIATIONS WITH WILD ANIMALS

When we understand land and water as given to more‐than‐human beings and insist that we ought to respect nonhuman animals and reciprocate the gifts of land and wild animal communities, then we enter into a new political ontology. As Coulthard (2014) suggested, this ontology is informed by land—conceived as a myriad of relationships between humans, wild animal communities, and other beings. Operating within such an ontology, Brian Noble (2018) explains that political treaties rest on an ecological ground.

Noble offers an example of how two tribes use their shared relationship with a broader ecological whole to negotiate jurisdiction. He explains that the Piikani and Ktunaxa communities had their separate territories and between them was a shared zone. Ktunaxa hunters were found transgressing this understanding as they had followed a community of black‐tailed deer through the shared space into the distinctly Piikani territory. The Ktunaxa had also performed a ceremony to aid in hunting these deer; through this ceremony, they took the deer to carry a powerful spirit and medicine.^10^ Respect for the deer entailed following them not just for meat but to follow their medicine. When the Piikani found them, the Ktunaxa admitted they were in Piikani territory and came to an agreement. They transferred the medicine to the Piikani; thereafter, both the Ktunaxa and Piikani would follow and hunt the black‐tailed deer where the deer would lead them (Noble, 2018, pp. 317–321). This relationship between the land, the deer, the Piikani, and the Ktunaxa is a complex system of relations involving respect for each other and the deer. In this case, the deer's autonomy was essential to negotiating how to share the land.

The deer were conceived as knowing the land, being free to move on it, and even leading the humans. The land is given to the deer, and *the* deer give themselves to the hunters. The Ktunaxa and Piikani understand that they depend on and respect the deer. This mutual respect for deer forms the common ground upon which they can negotiate flexible and mutually beneficial boundaries. Noble (2018) stresses that Indigenous understandings of how human communities and ecosystems relate are built on giving and not taking. The land gives vitality to human and wild animal communities. This gift is shared from the beginning; humans do not have privileged authority.

If we consider wild animals as having their own jurisdiction, which overlaps Piikani and Ktunaxa jurisdictions, we can see the deer's jurisdiction is also being respected here. The deer are not penned in, and humans are not giving themselves incentives to overhunt or control the migration of the deer. While, in this case, the deer were not explicitly represented in the negotiation, respect for the deer, including their freedom to move through *their* territory, was implicitly part of the human negotiations.

We might take this one step further and describe human communities as having treaties with wild animal communities. Simpson (Alderville First Nation, 2017) describes just such an interspecies agreement and suggests it is part of an underlying value for “Internationalism.” She describes her own experience as an Indigenous scholar, who relies on the teachings of many nations. Traveling between nations learning and respecting the similarities and differences in these practices is an important part of her experience.

Here, her use of “internationalism” has less to do with overlapping territory or international agreements and more to do with valuing the conversations that come from traveling between and living among multiple nations. This overlapping multiplicity is not exclusive to human nations. She tells the story of the Nishnaabeg^11^ Treaty with Hoof Nation. The deer, which the Nishnaabeg depend on, have left. Some people go out looking for them; when the Hoof Clan of deer are found, diplomats, spiritual people, and mediators listen to them.

After some negotiation, the people learned that the hoof Clan had left their territory because the Nishnaabeg were no longer honoring them. They had been wasting their meat and not treating their bodies with proper reverence. The Hoof Clan had withdrawn from their territory and their relationship with the Nishnaabeg. They had stopped participating. (Simpson, 2017, p. 61).

Having learned this, the Nishnaabeg offered to treat the Hoof Clan with proper respect, including providing the proper rituals associated with hunting. With this promise, the Hoof Clan returned to their territory. There are important lessons in this story about how we must respect wild animals in order for it to be possible to live with them. However, Simpsons' point is that this story characterizes the land as inhabited by many clans, including more‐than‐human nations like Hoof nation. In this sense, Nishnaabeg internationalism presents more‐than‐human political life as dependent on negotiating relationships with others. These negotiations illustrate how communities can share a space and live with each other, such that all involved communities are respected.

This sense of internationalism suggests human and wild animal communities have relationships that in some ways are best thought of as “between nations,” without presupposing exclusive, adversarial, and coercive powers. Instead, interspecies internationalism ought to presuppose an always already shared territory. This Grounded Authority is something that both human and wild animal communities possess, such that they must leave enough so other communities can “avoid not having enough.”

## 8 WILD ANIMALS WITH GROUNDED AUTHORITY

Representatives of Hoof nation describe the situation to the Nishnaabeg without appealing to the sovereignty of Hoof nation. Instead, they start with the deers' behavior. Hoof nation left, driven out by disrespectful human behavior. leaving is a political activity deer can do. The overlapping jurisdiction of Hoof nation is implicit in the acknowledgement that humans did wrong in driving them out.

This form of representation might better capture the aims of Donaldson and Kymlicka's (2011) Wild Animal Sovereignty than “sovereignty” does. Representatives should be able to advocate for wild animal communities by representing the activities of those communities. Behavior, like Hoof nation's leaving depleted or dangerous land, indicates their intolerance of disrespectful behavior by humans, and it was the behavior of Hoof nation that is described by the humans who represent them. The behavior of Hoof nation was what set a limit on how humans interact with them, in this way Hoof nation participates in a negotiation and that participation is then interpreted and represented. It is ontologically distinct from representing the interests of nonparticipants, even if it largely results in the same recommendations.

Meijer (2019) suggests that persistent conflicts with wild animals caused by their occupying a particular place or migrating are a plausible form of political communication. To better understand how animal behavior communicates, we might work with animal scientists toward coexistence that respects the agency and autonomy of wild animal communities (Caro & Sherman, 2013; Greggor et al., 2014; Santiago‐Ávila & Lynn, 2020).

Grounded Authority merely fills in a gap here. In order to understand the political implications of movements, behavior, and occupation of places by wild animal communities, we must understand those communities' as having political standing. A jurisdiction based on their shared receipt of and respect for the gifts of land provided such a standing. Let me offer an everyday example; I mentioned that a turtle crossing the road disrupted my normal view that roads are not for turtles. In that encounter, I moved the turtle to the side of the road it came from, the side with a lake where I presume she lives. Blanding's turtles in lake Scugog nest on higher ground and, therefore, must have access across roads to get from their lake to their nesting sites. I presumed that as the human I knew better, I moved her off the road but back to where she started, forcing her to cross the road again. I could have respected her agency and her knowledge of the land by recognizing roads are the sorts of things turtles are supposed to cross. As a community, we could take persistent road crossings as a sign from a wild animal community that the existing relationship is not working.

We might look to repair that relationship and better share the land we all need access to by building tunnels and fences to help creatures like the turtles safely cross major roads. Such projects are already underway to protect Blanding's and other turtle and amphibian species (Boyle et al., 2021; Longwell, 2021; Markle et al., 2017). The representatives we need are already here; they are often activists. Understanding the jurisdiction of wild animals shows that activists and environmental agencies could represent shared jurisdiction by attending to wild animal behavior. Wild animal behavior, like crossing roads, offers a site for us to negotiate how to share. The land is theirs already, given to them by the land; we must respect that.

## CONFLICT OF INTEREST

The author declares that there is no conflict of interest that could be perceived as prejudicing the impartiality of the research reported.

## Endnotes

^1^
While this group is very diverse, and I will be leaning on several distinct traditions, they share some similarities that can decenter and challenge the status quo western political ontology manifest in the Settler Canadian and U.S. governments.

^2^
I use “More‐than‐human beings” to describe the inhabitants of the more‐than‐human world, this includes humans, nonhuman animals, and other animate beings like plants, rivers, and spirits.

^3^
Wild Animal Sovereignty has been capitalized to distinguish it from Westphalian sovereignty of wild animals, described by Goodin et al. (1997).

^4^
In other contexts, Grounded Authority or similar forms of jurisdiction might be described as forms of sovereignty because, like Wild Animal Sovereignty, it is a form of jurisdiction that participates in an international community. However, it is distinct from Westphalian sovereignty.

^5^
Much of the land Settler Canada controls remains unceded territory, not governed by treaties.

^6^
Borrows is specifically thinking of the Mississauga dialect of Anishinaabemowin, which is closely related to and often mutually intelligible with the Algonquin language.

^7^
Mississaugas are an Anishinaabe people, who are the treaty holders of the Toronto Purchase Treaty 13, and other treaties and land claims in Southern Ontario.

^8^
Coulthard specifies that this is true at least in the Weledeh dialect.

^9^
The Mi'kmaq are a First Nations people whose traditional territory includes Atlantic Canada, part of Quebec and Maine.

^10^
“Medicine,” in this case, is not material used in treating ailments but a different contribution to health.

^11^
This is another term for the Anishinaabe, Simpson is specifically referring to the Mississaugas.

## References

[josp12498-bib-0001] Alfred, Taiaiake . 2006. “Sovereignty—An Inappropriate Concept.” In The Indigenous Experience: Global Perspectives, edited by Roger Maaka and Chris Anderson , 332–6. Toronto: Canadian Scholars' Press.

[josp12498-bib-0002] Ball, David . 2021. “Judge Says Rcmp Acted ‘Unlawfully’ Enforcing Fairy Creek Injunction after Court Challenge.” CBC News, August 13, 2021. https://www.cbc.ca/news/canada/british-columbia/rcmp-fairy-creek-court-case-1.6139351

[josp12498-bib-0003] Borrows, John . 2018. “Earth‐Bound: Indigenous Resurgence and Environmental Reconciliation.” In Resurgence and Reconciliation, edited by Michael Asch and John Borrows , 60–92. Toronto: University of Toronto Press.

[josp12498-bib-0004] Boyce, Mark S. 2018. “Wolves for Yellowstone: Dynamics in Time and Space.” Journal of Mammalogy 99(5): 1021–31.

[josp12498-bib-0005] Boyle, Sean P. , M. G. Keevil , Jacqueline D. Litzgus , Don Tyerman , and David Lesbarrères . 2021. “Road‐Effect Mitigation Promotes Connectivity and Reduces Mortality at the Population‐Level.” Biological Conservation 261: 109230.

[josp12498-bib-0006] Brakes, Philippa , Emma L. Carroll , Sasha R. X. Dall , Sally Keith , Peter K. McGregor , Sarah L. Mesnick , Michael J. Noad , et al. 2021. “A Deepening Understanding of Animal Culture Suggests Lessons for Conservation.” Proceedings of the Royal Society B: Biological Sciences 288(1949): 20202718. 10.1098/rspb.2020.2718 PMC8059593 33878919

[josp12498-bib-0007] Callicott, J. Baird . 1982. “Traditional American Indian and Western European Attitudes toward Nature: An Overview.” Environmental Ethics 22: 293–318.

[josp12498-bib-0008] Callicott, J. Baird . 2000. “Many Indigenous Worlds or the Indigenous World? A Reply to my ‘Indigenous’ Critics.” Environmental Ethics 22: 291–310.

[josp12498-bib-0009] Caro, Tim , and Paul W. Sherman . 2013. “Eighteen Reasons Animal Behaviourists Avoid Involvement in Conservation.” Animal Behaviour 85(2): 305–12.

[josp12498-bib-0010] Cochrane, Alasdair , Robert Garner , and Siobhan O'Sullivan . 2018. “Animal Ethics and the Political.” Critical Review of International Social and Political Philosophy 21(2): 261–77.

[josp12498-bib-0011] Cochrane, Alastair . 2013. “Cosmozoopolis: The Case against Group‐Differentiated Animal Rights.” Law, Ethics and Philosophy 1: 127–41.

[josp12498-bib-0012] Coulthard, Glen . 2014. Red Skin, White Masks: Rejecting the Colonial Politics of Recognition. Minneapolis: University of Minnesota Press.

[josp12498-bib-0013] Donaldson, Sue . 2020. “Animals and Citizenship.” Minding Nature 13(2): 22–7.

[josp12498-bib-0014] Donaldson, Sue , and Will Kymlicka . 2011. Zoopolis: A Political Theory of Animal Rights. Oxford: Oxford University Press.

[josp12498-bib-0015] Drinkwater, Rob . 2021. “Iqaluit Community Bands Together as Drinking Water Remains Contaminated by Fuel." Global News, October 18, 2021. globalnews.ca/news/8273375/iqaluit-community-bands-drinking-water-contaminated-fuel/

[josp12498-bib-0016] Fligg, Robert A. , and Derek T. Robinson . 2020. “Reviewing First Nation Land Management Regimes in Canada and Exploring their Relationship to Community Well‐Being.” Land Use Policy 90: 104245.

[josp12498-bib-0017] Goodin, Robert , Carole Pateman , and Roy Pateman . 1997. “Simian Sovereignty.” Political Theory 25(6): 821–49.

[josp12498-bib-0018] Greggor, Alison L. , Nicola S. Clayton , Ben Phalan , and Alex Thornton . 2014. “Comparative Cognition for Conservationists.” Trends in Ecology & Evolution 29(9): 489–95. 25043737 10.1016/j.tree.2014.06.004 PMC4153814

[josp12498-bib-0019] Hadley, John . 2005. “Nonhuman Animal Property: Reconciling Environmentalism and Animal Rights.” Journal of Social Philosophy 3(36): 305–15.

[josp12498-bib-0020] Kimmerer, Robin W. 2013. Braiding Sweetgrass. Minneapolis: Milkweed Editions.

[josp12498-bib-0021] Kymlicka, Will , and Sue Donaldson . 2014. “Animal Rights and Multiculturalism, and the Left.” Journal of Social Philosophy 45(1): 116–35.

[josp12498-bib-0022] Kymlicka, Will , and Sue Donaldson . 2015. “Animal Rights and Aboriginal Rights.” In Canadian Perspectives on Animals and the Law, edited by Peter Sankoff , Vaughan Black , and Katie Sykes , 159–86. Toronto: Irwin law Inc.

[josp12498-bib-0023] Larsen, Karin . 2021. “Fairy Creek Protest on Vancouver Island Now Considered Largest Act of Civil Disobedience in Canadian History.” CBC News, September 9, 2021. https://www.cbc.ca/news/canada/british-columbia/fairy-creek-protest-largest-act-of-civil-disobedience-1.6168210

[josp12498-bib-0024] Leopold, Aldo . 1949. A Sand County Almanac. Oxford: Oxford University Press.

[josp12498-bib-0025] Longwell, Karen . 2021. “Brampton Built Crossing Tunnels for Wildlife Under Local Roads.” BlogTO, January 25, 2021. https://www.blogto.com/city/2021/01/brampton-built-crossing-tunnels-wildlife-under-local-roads/

[josp12498-bib-0026] Markle, Chantel E. , Scott D. Gillingwater , Rick Levick , and Patricia Chow‐Fraser . 2017. “The True Cost of Partial Fencing: Evaluating Strategies to Reduce Reptile Road Mortality.” Wildlife Society Bulletin 41(2): 342–50.

[josp12498-bib-0027] McKinley, Steve , and Alex McKeen . 2020. “‘The RCMP Just Stood there’: Attack on Mi'kmaq Fishery Sparks Tense Standoff Condemnation.” Toronto Star, October 14, 2020. https://www.thestar.com/news/canada/2020/10/14/mikmaq-chief-slams-nova-scotia-fishery-violence-they-are-getting-away-with-these-terrorist-hate-crime-acts.html

[josp12498-bib-0028] Meijer, Eva . 2019. When Animals Speak: Toward an Interspecies Democracy. New York: New York University Press.

[josp12498-bib-0029] Nibert, David . 2002. Animal Rights/Human Rights: Entanglements of Oppression and Liberation. Plymouth: Rowman & Littlefield Publishers.

[josp12498-bib-0030] Noble, Brian . 2018. “Treaty Ecologies: With Persons, Peoples, Animals, and the Land.” In Resurgence and Reconciliation, edited by Michael Asch and John Borrows , 326–53. Toronto: University of Toronto Press.

[josp12498-bib-0031] Nussbaum, Martha . 2006. Frontiers of Justice: Disability, Nationality, Species Membership. Cambridge, MA: Harvard University Press.

[josp12498-bib-0032] Pasternak, Shiri . 2017. Grounded Authority: The Algonquins of Barriere Lake against the State. Minneapolis, MN: University of Minnesota Press.

[josp12498-bib-0033] Regan, Tom . 1985. “The Case for Animal Rights.” In In Defence of Animals, edited by Peter Singer , 13–26. Oxford: Basil Blackwell.

[josp12498-bib-0034] Robinson, Margaret . 2014. “Animal Personhood in Mi'kmaq Perspective.” Societies 4: 672–88.

[josp12498-bib-0035] Rolston, Holmes . 1988. Environmental Ethics: Duties to and Values in the Natural World 1988. Philadelphia: Temple University Press.

[josp12498-bib-0036] Sainato, Michael . 2021. “Protesters Against Line 3 Tar Sands Pipeline Face Arrests and Rubber Bullets.” The Guardian, August 10, 2021. https://www.theguardian.com/environment/2021/aug/10/protesters-line-3-minnesota-oil-gas-pipeline

[josp12498-bib-0037] Santiago‐Ávila, Francisco , and William Lynn . 2020. “Bridging Compassion and Justice in Conservation Ethics.” Biological Conservation 248: 108648.

[josp12498-bib-0038] Simpson, Leanne . 2017. As We Have Always Done: Indigenous Freedom through Radical Resistance. Minneapolis: University of Minnesota Press.

[josp12498-bib-0039] Singer, Peter . 1974. “All Animals Are Equal.” Philosophic Exchange 1(5): 103–16.

[josp12498-bib-0040] Van Schie, Rosanne , and Wolfgang Haider . 2015. “Indigenous‐Based Approaches to Territorial Conservation: A Case Study of the Algonquin Nation of Wolf Lake.” Conservation and Society 13(1): 72–83.

[josp12498-bib-0041] Wadiwel, Dinesh . 2015. The War against Animals. Leiden: Brill Rodopi.

